# The Antimicrobial and Antioxidant Effects of Bilayer Films Based on Polylactic Acid (PLA)/Chitosan: Starch Containing Bitter Orange Essential Oil on the Fresh Rainbow Trout (*Onchorhynchus mykiss*) Fillet

**DOI:** 10.1002/fsn3.70139

**Published:** 2025-04-16

**Authors:** Hossein Nourani, Seyed Mahdi Ojagh, Masoud Rezaei, Alireza Alishahi, Shahab Naghdi

**Affiliations:** ^1^ Gorgan University of Agricultural Sciences and Natural Resources Gorgan Iran; ^2^ Department of Fisheries, Faculty of Natural Resources University of Tehran Karaj Iran; ^3^ Department of Fisheries, Faculty of Natural Resources and Marine Sciences Tarbiat Modares University Noor Iran

**Keywords:** active packaging, bitter orange essential oil, chitosan, polylactic acid, rainbow trout, starch

## Abstract

This study investigated the antibacterial properties of polylactic acid (PLA)/chitosan: starch films incorporated with bitter orange essential oil (BOEO) for preserving rainbow trout fillets. Three BOEO concentrations (0.7%, 1.4%, and 2.1% w/w) were incorporated via casting. Increased BOEO concentration enhanced film hydrophobicity and thermal resistance, but reduced tensile strength while increasing elongation. Atomic force microscopy revealed altered surface roughness. Antibacterial testing showed optimal activity against both Gram‐positive and Gram‐negative bacteria at 1.2% BOEO. Films containing 1.2% BOEO were applied to rainbow trout fillets, significantly reducing spoilage bacteria (total bacterial count, psychrotrophic, lactic acid bacteria, and Enterobacteriaceae), slowing chemical spoilage (pH and TBA values), and minimizing weight loss during 16 days of refrigerated storage (4°C). These results demonstrate the potential of BOEO‐incorporated PLA/chitosan: starch films for extending the shelf life of rainbow trout.

## Graphical abstract

The study demonstrated that bilayer films based on polylactic acid (PLA)/chitosan: starch containing bitter orange essential oil (BOEO) effectively inhibited spoilage bacteria in rainbow trout fillets during refrigerated storage. Films with 1.2% BOEO significantly reduced bacterial counts and slowed chemical spoilage, maintaining quality over 16 days. Additionally, increased BOEO concentration enhanced film properties such as hydrophobicity and thermal resistance, highlighting the potential of these films for extending the shelf life of fish products.

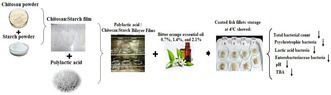

## 1 Introduction

Consumers in the food industry have a high demand for fresh meat products, which are generally well‐received (Nasution et al. 2023). However, the short shelf life of these products is a concern for food companies. Fish, such as *Oncorhynchus mykiss* , being a valuable source of protein, has the ability to provide a significant portion of the protein required by humans due to the wide variety of species available (Naghdi et al. 2023). Seafood products quickly lose their quality due to various chemical, enzymatic, and physical changes that directly affect the four main categories of compounds in these products: proteins, fats, carbohydrates, and moisture (Far et al. 2023). These changes include alterations in amino acids, enzymes, and nitrogenous bases such as ammonia and TMAO (Far et al. 2023). Changes in the taste and aroma of seafood products occur rapidly due to oxidation reactions and contamination with microorganisms. Improving storage methods can enhance the quality and shelf life of these products, thereby preventing customer dissatisfaction and market loss (Kumar et al. 2023).

Packaging plays a crucial role in the preservation and marketing of food products. It serves as the first point of contact between consumers and products, influencing the perceived quality of the goods (Zeid et al. 2019). New packaging technologies are being developed to meet consumer demands for fresh, tasty products with extended shelf life and controlled quality (Zeid et al. 2019). Changes in consumer behavior and retail practices pose significant challenges to the food packaging industry, driving the development of innovative packaging concepts to enhance preservation, quality control, and food safety (Sreekanth et al. 2024). The use of petroleum‐based packaging has raised considerable environmental concerns among consumers (Sreekanth et al. 2024). Consequently, in recent years, both industry and consumers have shifted towards producing and utilizing biodegradable polymers as an alternative solution (Wang et al. 2022). Biodegradable polymers are those that can be degraded under aerobic and anaerobic conditions, converting into materials that are capable of returning to the soil (Kumar et al. 2023). Biopolymers can be classified based on their origin into categories animal‐based, plant‐based, polymers derived from microorganisms, lipids (animal and plant‐based), polysaccharides (such as cellulose, starch, and chitin), proteins (like corn zein, wheat gluten, and soy protein), and natural monomers (Zhang et al. 2021). Today, biodegradable polymers sourced from renewable sources such as starch and chitosan help conserve natural resources, are environmentally friendly, recyclable, and have attracted significant attention from researchers (Zhang et al. 2021). They have great potential in packaging applications. Also, Polylactic acid (PLA) is known as a biodegradable, heat‐moldable, and renewable polymer, which offers numerous advantages including significant energy savings, the ability to recycle lactic acid through hydrolysis or alcoholysis, the potential for producing compostable paper‐plastic composite packaging, waste volume reduction, agricultural benefits, and the ability to modify its physical properties (Wang et al. 2022). Active packaging, a novel concept in food packaging, responds to evolving consumer needs and market trends by incorporating chemical or biological changes within the packaging to improve food safety and shelf life (Chen et al. 2024). The use of active packaging can offer practical benefits in food preservation. Antimicrobial packaging with gradual or controlled release of antimicrobial compounds into food helps prevent their spoilage (Zeid et al. 2019). Generally, the antimicrobial agents used in AFCMs consist of organic, mineral, and bioactive compounds, with a preference for known natural and non‐toxic antimicrobial agents due to environmental and health concerns (Kamkar et al. 2021; Song et al. 2022). Plant‐based essential oils effectively kill many harmful microbes and are considered safe for use as Generally Recognized as Safe (GRAS) by the FDA. Among the various essential oils, bitter orange essential oil extracted from the plant *Citrus aurantium* is well‐known for its antimicrobial and antioxidant properties (Gaff et al. 2020). These activities are primarily attributed to two phenolic groups, carvacrol and thymol, and the monoterpene hydrocarbons p‐cymene and γ‐terpinene (which are present in lower concentrations) (Gaff et al. 2020; Mejri et al. 2024). Bitter orange essential oil is considered a promising candidate for developing antimicrobial agents in food because of its effective performance against harmful Gram‐positive and Gram‐negative bacteria (Mejri et al. 2024).

Therefore, the first phase of the current study focuses on constructing a two‐layer composite system of starch: chitosan/PLA. Based on the information provided, the objective of the current study is to prepare a two‐layer film based on starch: chitosan/polylactic acid containing bitter orange essential oil. Then, this film was used to store rainbow trout fillets in the refrigerator.

## 2 Material and Methods

### 2.1 Preparation of PLA Film

The stages of this research were carried out in the Processing Laboratory of Tarbiat Modares University. The PLA (bought from the Sigma) film was prepared using the molding method. Initially, polylactic acid granules were dried for 3 h in an oven at 105°C. Subsequently, after cooling in a desiccator, a 5:1 weight‐to‐weight (w/w) solution of PLA in chloroform was placed on a magnetic stirrer (speed 400 rpm) at 25°C for 1 h. The polymer solution was then poured into a Petri dish and left to dry at room temperature for 3 days. After drying, the films were gently separated from the containers and sealed in plastic bags before analysis (Jamshidian et al. 2010).

### 2.2 Preparation of Chitosan/Starch Films

To produce two‐layer chitosan/starch films, solutions of 2% chitosan and 3% starch (bought from Sigma‐Aldrich (St. Louis, MO, USA)) were first prepared. For the chitosan solution preparation, 2 g of chitosan powder were dissolved in 100 mL of 1% acetic acid that had been prepared in advance, and the solution was placed on a magnetic stirrer at room temperature for 24 h. Subsequently, the prepared chitosan solution was transferred to a Falcon tube and centrifuged at 9000 rpm for 20 min (Fan et al. 2023; Istiqomah et al. 2022). Next, for the preparation of the starch solution, 3 g of starch powder were dissolved in 100 mL of distilled water at 80°C for 24 h on a magnetic stirrer. Then, the prepared chitosan and starch solutions were mixed in specified volume‐to‐volume ratios according to Table 1 to form a two‐layer film solution. Glycerol (0.3 g/g dry matter) was added to the film‐forming solution and stirred for an additional 15 min at 45°C. Subsequently, to remove bubbles within the polymer matrix, a bubble removal process was carried out for 15 min. Finally, a certain amount of the prepared solutions was poured into plastic containers and placed in an oven at 45°C for 48 h to form composite films. After drying, the films were gently separated from the containers and sealed in plastic bags for storage and further testing.

**TABLE 1:** Composition of two‐part starch/chitosan films.

Film	Starch 3%	Chitosan 2%
Starch/Chitosan	100	0
Starch/Chitosan	75	25
Starch/Chitosan	50	50
Starch/Chitosan	25	75
Starch/Chitosan	0	100

### 2.3 Preparation of Two‐Layer Film Based on PLA‐Starch/Chitosan Containing Bitter Orange Essential Oil

A specific volume of the optimized two‐layer starch/chitosan film‐forming (treatment 25/75% starch/chitosan), as shown in Table 2, was poured onto the first layer (dried PLA film) and placed in an oven at 45°C for 24 h for drying. After drying, the films were gently separated from the containers and sealed in plastic bags for storage and further testing. Additionally, to prepare a two‐layer film containing bitter orange, after conducting physical/mechanical tests and selecting the optimal ratio of PLA‐starch/chitosan, a two‐layer film‐forming solution containing bitter orange at three levels (BOEO) (0.7%, 0.4%, 0.2%) previously prepared was poured onto the first layer (dried PLA film) in a specific ratio to the PLA solution and dried in the oven at 45°C for 24 h.

**TABLE 2:** Composition of two‐layer films based on PLA and starch‐chitosan composite containing bitter orange essential oil.

Film	Starch‐Chitosan	PLA
Starch/Chitosan‐PLA	100	0
Starch/Chitosan‐PLA	75	25
Starch/Chitosan‐PLA	50	50
Starch/Chitosan‐PLA	25	75
Starch/Chitosan‐PLA	0	100

Film	Bitter orange essential oil (BOEO) %	Starch/Chitosan‐PLA
Starch/Chitosan‐PLA‐BOEO	0.7	Optimal ratio of PLA‐starch/chitosan
Starch/Chitosan‐PLA‐BOEO	1.4	Optimal ratio of PLA‐starch/chitosan
Starch/Chitosan‐PLA‐BOEO	2.1	Optimal ratio of PLA‐starch/chitosan

### 2.4 Characterization of the Prepared Films

#### 2.4.1 Measurement of Film Thickness and the Contact Angle of Films

Using a digital micrometer (0.01 mm accuracy), the thickness of each sample was measured at seven points. The average thickness determined the tensile strength and water vapor permeability. To measure the contact angle of the films, the Sessile drop method was used. For this purpose, 5 μL of double distilled water was placed on the samples by the device, and the contact angle of the droplet with the film was reported at the initial time (Javidi et al. 2016).

#### 2.4.2 Mechanical Properties Determination of Films

To assess the mechanical properties of the films, factors such as tensile strength (TS) in MPa, percent elongation at break (EAB), and modulus of elasticity (EM) in GPa were measured according to ASTM D88‐02 standard (ASTM, 2002). Initially, the samples were equilibrated at 55% humidity in a desiccator containing magnesium nitrate for 48 h. Subsequently, three samples from each treatment, measuring 10 × 2 cm, were cut and placed between the jaws of the machine with an initial distance of 3 cm and an upper jaw speed of 50 mm/min. The stress and strain values were then calculated from the obtained graphs and tables.

#### 2.4.3 Water Vapor Permeability of Films

This test was conducted according to the ASTM E96/E96M‐05 standard method. During this test, the produced films were sealed with silicone grease onto a glass cup with a 49 mm diameter containing 6 mL of distilled water (100% relative humidity). The cups were then placed inside a desiccator containing silica gel (approximately 0% relative humidity) at room temperature and allowed for the films to equilibrate for 2 h before weighing the cups. Subsequently, the cups were weighed at 2‐h intervals over a period of 10 h (Sarhadi et al. 2024).

#### 2.4.4 Water Absorption of Films

The moisture absorption of the films was measured using the method described by Sreekanth et al. (2024) at a temperature of 25°C ± 2°C over a period of 6 weeks. Initially, samples of the film were weighed using a digital balance with an accuracy of 0.0001 g, and their initial dry weight was calculated. The samples were then placed in an oven at 105°C and re‐weighed. Subsequently, the film samples were placed in sealed containers containing 30 mL of distilled water. After each 7‐day period, the samples were removed from the containers to dry off the excess water using blotting paper, and then re‐weighed before being returned to the containers with distilled water. The moisture absorption of the films over time was calculated as the average of three repetitions using the formula:

$$\text{Moisture Absorption}\;\left(\%\right)=100\times \left(\text{Initial Film Weight}/\text{Film Weight after immersion}-\text{Initial Film Weight}\right)$$

#### 2.4.5 Amount of Weight Loss of Films

Weight loss, determined according to Erdohan et al. (2013), was calculated as the percentage decrease in film weight after drying small pieces at 105°C for 24 h.

#### 2.4.6 Measurement of Surface Color, Turbidity, and the Amount of Light Passing Through Films

**FIGURE 1:**
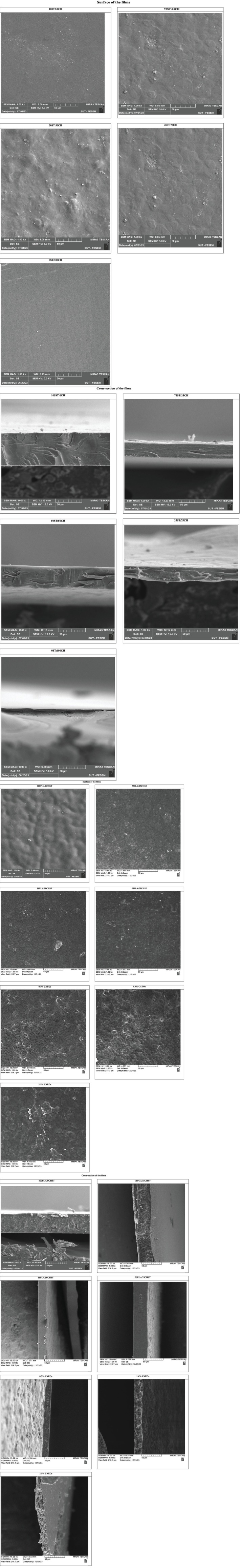
(a) Scanning Electron Microscope (SEM) images of the surface (left side of each treatment) and cross‐section (right side of each treatment) of chitosan:Starch composite films. (b) Scanning Electron Microscope (SEM) images of the surface (left picture of each treatment) and cross‐section (right picture of each treatment) of the polylactic acid/chitosan:Starch bilayer films and polylactic acid/chitosan:Starch films containing concentrations of 0.7%, 1.4%, and 2.1% BOEO.

**FIGURE 2:**
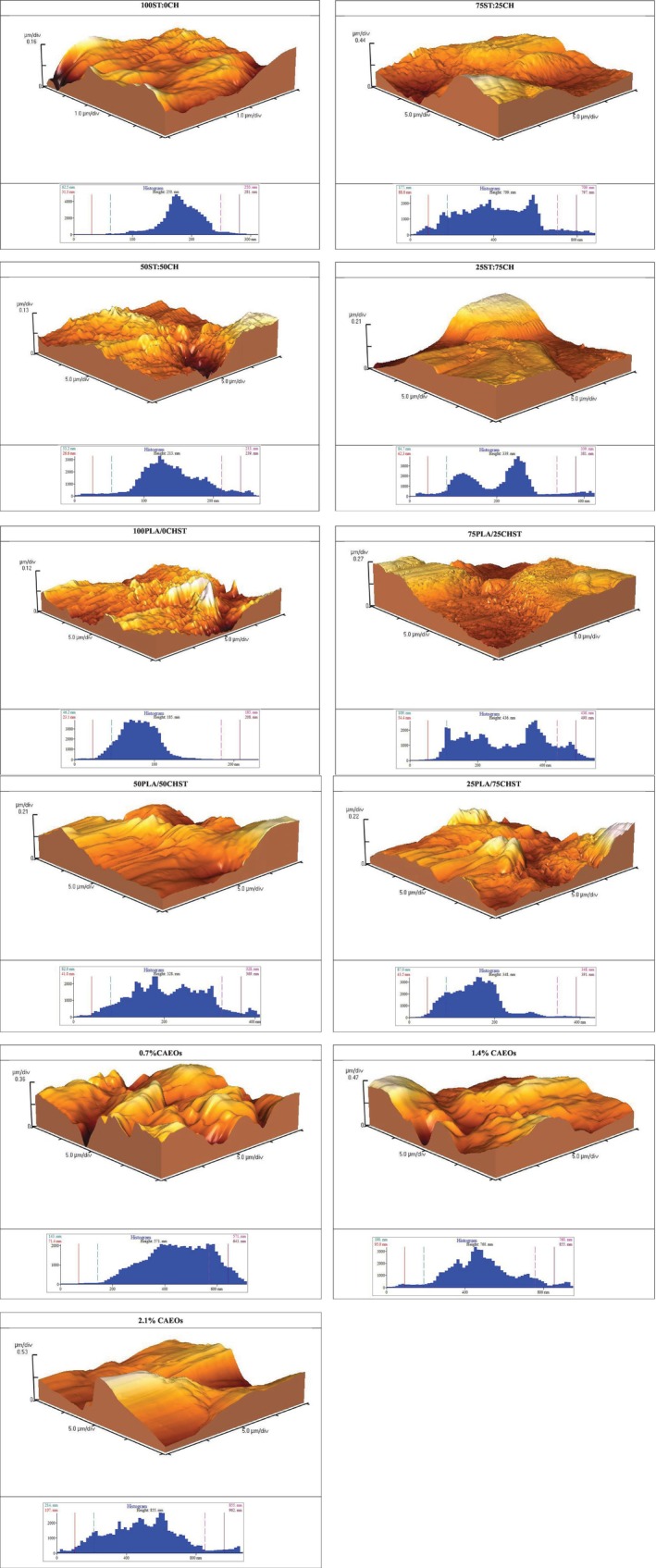
AFM images of the chitosan:Starch composite films and polylactic acid/chitosan:Starch bilayer films, and polylactic acid/chitosan:Starch films containing concentrations of 0.7%, 1.4%, and 2.1% BOEO.

**FIGURE 3:**
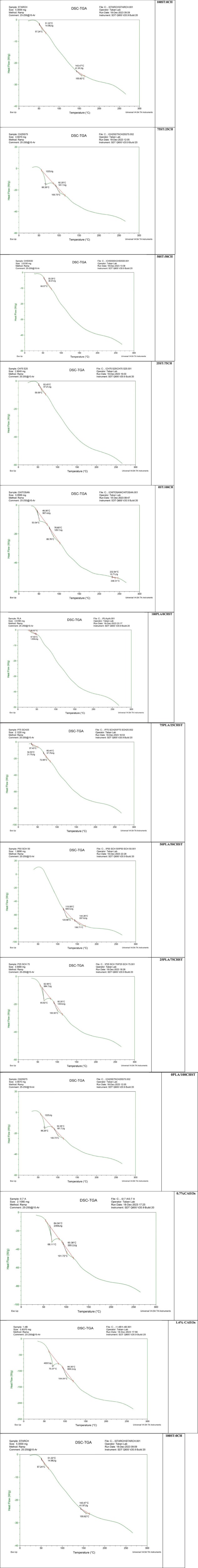
Differential scanning calorimeter (DSC) of the chitosan:Starch composite films and polylactic acid/chitosan:Starch bilayer films, and polylactic acid/chitosan:Starch films containing concentrations of 0.7%, 1.4%, and 2.1% BOEO.

Surface color, expressed as L, a, and b values, was determined using a standard white tile (L* = 63.94, a* = 0, b* = 65.0) as a background (Park et al. 2022). The a* and b* factors represent the red‐green and yellow‐blue axes, respectively (Figures 1, 2, 3). The color difference of the samples is compared using the ΔE factor.

### 2.5 Scanning Electron Microscopy (SEM) of Films

To examine the effects of adding bitter orange essential oil on the microstructure of the produced films, surface and cross‐sectional images of the films were prepared. For this purpose, dried pieces of the film were attached to aluminum holders using double‐sided adhesive. Subsequently, to enhance conductivity, a thin layer of gold was sputtered onto the films, and then they were placed inside a scanning electron microscope. Finally, imaging of the samples was carried out at various magnifications to analyze the microstructure of the films (Kamkar et al. 2021).

### 2.6 Atomic Force Microscopy (AFM) of Films

The atomic force microscopy test was used to describe the surface structure and measure the roughness of the two‐layer and double‐layer films containing 0.7%, 0.4%, and 0.2% bitter orange essential oil. For image registration, a triangular‐based silicon probe with a force constant of 50 nanoNewtons was used.

### 2.7 Differential Scanning Calorimetry (DSC) Analysis of Films

To investigate the thermal behavior of the prepared films, a differential scanning calorimetry (DSC) instrument was used. Approximately 10 mg of sample was placed inside aluminum cells. The samples were scanned at a rate of approximately 10°C/min. The thermal cycle used for each sample covered a temperature range from 25°C to 250°C under a constant flow of nitrogen atmosphere. The melting point (Tm) and glass transition temperature (Tg) were determined from the temperature curves (Javidi et al. 2016).

### 2.8 FT‐IR Spectroscopy of Films

To investigate the chemical bonds of the materials constituting the films, the produced films were analyzed using an Attenuated Total Reflectance (ATR) Fourier Transform Infrared (FTIR) spectrometer (Zomorodian et al. 2023). Film samples were dried in a silica gel desiccator for 7 days before analysis. Subsequently, pieces of the film with a diameter of 2 cm were placed between two KBr windows. The ATR spectra were recorded in the wavenumber range of 400 to 4000 cm^−1^.

### 2.9 Assessment of Antioxidant Properties of Produced Films

Antioxidant activity was assessed using the DPPH free radical scavenging assay (Kamkar et al. 2021). Briefly, 3 mg of film was mixed with 3 mL methanol for 5 min. 1 mL of this extract was then added to 1 mL of 1 mM DPPH in methanol, incubated at room temperature for 30 min, and the absorbance at 517 nm was measured using a spectrophotometer. The percentage DPPH scavenging activity was calculated using the formula of Williams et al. (1995):

$$I=\left(A\_\text{blank}-A\_\text{sample}\right)/A\_\text{blank}\times 100$$

In this formula, A_blank represents the optical absorption of the negative control lacking the film (methanolic DPPH solution), and A_sample indicates the optical density of various concentrations of the film extract.

### 2.10 Evaluation of the Antimicrobial Effects of BOEO and Production Films

To evaluate the antimicrobial activity of the oil, the disk and well diffusion tests were conducted using an agar medium. After preparing bacterial strains (CFU/ml 10^8^), a surface culture was performed using a sterile swab with 1.0 mL of liquid culture containing 10^6^ bacteria of interest (*Listeria monocytogenes, Escherichia coli, Salmonella enteritidis, Staphylococcus aureus*) on Tryptone Soy Agar culture medium. Inoculated plates were used for both the disk and well diffusion tests.

For the well diffusion test, wells with a diameter of 6.0 cm were created using sterile pipettes (1 cc), and then the ends of the wells were sealed with Tryptone Soy Agar culture medium. After drying under a microbiological hood, 40 μL of the oil of interest was added to the wells.

For the disk diffusion test, 10 μL of BOEO were poured onto sterile paper disks with a diameter of 6 mm. After drying, the disks were transferred to the culture medium containing bacteria under sterile conditions. In each plate, 3 to 4 disks were placed equidistant from each other. The plates were then incubated at 37°C in an incubator for 24 h. After incubation, the bacterial growth was evaluated by measuring the diameter of the formed inhibition zones, indicating the antimicrobial efficacy. To ensure uniform bacterial growth on the plate surfaces, a plate without disks and wells was included for each tested bacterium. Additionally, a plate without bacteria was used to ensure the sterility of the culture environments (Gómez‐Estaca et al. 2010).

To assess the antimicrobial properties of the films made, the direct contact method was utilized. Firstly, sterile film samples with a diameter of 5.1 cm were prepared and placed on Tryptone Soy Agar culture medium containing 10^6^ bacteria and on plates containing Tryptone Soy Agar culture medium with 10^6^ bacteria, following the method by Atef et al. (2019). Subsequently, all plates were incubated at 37°C in an incubator for 24 h. After the incubation period, the bacterial growth was evaluated, and to ensure uniform bacterial growth on the plate surfaces, a plate without the film was included for each tested bacterium. Additionally, a plate without bacteria was used to confirm the sterility of the culture environments.

### 2.11 Evaluation of Antimicrobial Effects of Films on Fish Fillets

#### 2.11.1 Fish Preparation

Rainbow trout fish with an average weight of 300–400 g and an average length of 30 cm were purchased from a fish farm in Noor County. After gutting and scaling, fillets were prepared and cut into 2 × 2 cm pieces. After thorough washing, the pieces were placed under a microbiological hood to ensure that the fillets were free of excess water.

#### 2.11.2 Preparation of Antimicrobial Films

As mentioned in the introduction, the goal of this research was to prepare biodegradable films with suitable antimicrobial efficacy to increase the shelf life of rainbow trout fish fillets during cold storage. After preparing biodegradable films containing different amounts of bitter orange oil and assessing their physical, mechanical, and thermal properties, the best films that exhibited acceptable characteristics both physically, mechanically, and antimicrobially were selected. Antimicrobial films with 1.2% BOEO were chosen to be used as coatings on rainbow trout fish fillets during the storage period in the refrigerator. The cut fish fillets (approximately 2 × 2 cm) were coated with the produced films, and to measure the microbial load and chemical changes, they were stored in the refrigerator at 4°C for 16 days. Evaluations were conducted every 4 days during this storage period.

### 2.12 Evaluation of Bacterial Load in Rainbow Trout Fish Fillets

A quantity of 5 g of rainbow trout fish fillet sample was homogenized with 45 mL of physiological saline solution under sterile conditions until uniform. Subsequently, appropriate dilutions were prepared, and 1 mL of the homogenates was used for culturing Enterobacteriaceae (cultured in duplicate under anaerobic conditions), lactic acid bacteria (cultured in duplicate under anaerobic conditions), psychrotrophic bacteria, and total bacteria using the pour plate method in Violet Red Bile Dextrose Agar (VRBD), MacConkey Agar (MRSA), and Tryptone Soy Agar (TSA) media, respectively (Sayyari et al. 2021). The enumeration of psychrotrophic bacteria and total bacterial load was conducted at 7°C for 10 days and at 30°C for 2 days, respectively. The enumeration of lactic acid bacteria was carried out at 30°C for 3 days, and the enumeration of Enterobacteriaceae was performed at 30°C for 2 days (Gómez‐Estaca et al. 2010).

### 2.13 pH And TBA Measurement of Fish Fillet During Storage

Tissue samples (5 g each) were homogenized in 50 mL of distilled water, and their pH was determined using a digital pH meter.

The TBA measurement was conducted using a colorimetric technique. A sample of 200 mg of minced fish was added to a 25‐mL flask and then diluted with 1‐butanol to the mark. A 5 mL portion of this solution was transferred to dry, stoppered tubes, followed by the addition of 5 mL of TBA reagent (which was prepared by dissolving 200 mg of TBA in 100 mL of 1‐butanol after filtration). The sealed tubes were then placed in a water bath at 95°C for 2 h before being cooled to room temperature. Finally, the absorbance at 532 nm was measured against a distilled water blank, and the TBA value (in milligrams of malondialdehyde per kilogram of fish tissue) was calculated using the specified equation: TBA = ((As − Ab) × 50)/200.

### 2.14 Data Analysis

To draw the graphs in this study, Sigma Plot 12 and Origin 8.5 software were used. Additionally, statistical analysis of the data was performed using Excel and SPSS 16 software. Initially, the normality of the data was assessed in SPSS using the Kolmogorov–Smirnov test, followed by checking the homogeneity of variance using the Levene test. One‐way analysis of variance (ANOVA) with Dunnett's post hoc test was used to statistically compare the film properties and analyze the quantitative values obtained from microbial analyses. All results from three replicates were presented as mean ± standard deviation, and statistical comparisons were conducted at a confidence level of 95%.

## 3 Results and Discussion

### 3.1 Mechanical Properties Thickness, Tensile Strength, Elongation

As observed in Table 3, the thickness of bilayer films produced by adding chitosan significantly decreased compared to the control film. Additionally, while adding chitosan increased the film's elongation at break, it decreased at the moment of rupture, and tensile strength increased. The addition of BOEO resulted in increasing the thickness of the bilayer films compared to the control sample, which can be attributed to the entrapment of microdroplets of BOEO in the bilayer films. Furthermore, adding bitter orange oil led to a decline in the elongation at break and a decrease in its tensile strength, which could be due to the creation of a heterogeneous and discontinuous structure in these films. Li et al. (2019) revealed that adding turmeric essential oil into the chitosan matrix led to a decline in TS of the prepared film. In another study, using ginger essential oil (GEO) in a chitosan‐based film resulted in decreasing the TS (Al‐Ali et al., 2021).

**TABLE 3:** The results of thickness, tensile strength, and elongation percentage of bilayer PLA‐chitosan/starch films containing BOEO.

Treatments	Thickness (mm)	Elongation at break (%)	Tensile strength (MPa)
100PLA/0CHST	0.059 ± 0.05^c^	2.64 ± 0.23^c^	46.72 ± 2.82^a^
75PLA/25CHST	0.038 ± 0.005^d^	2.07 ± 0.39^c^	46.63 ± 2.80^a^
50PLA/50CHST	0.041 ± 0.006^d^	9.32 ± 0.44^b^	48.53 ± 0.13^a^
25PLA/75CHST	0.058 ± 0.007^c^	31.78 ± 1.62^a^	23.34 ± 1.83^c^
0PLA/100CHST	0.060 ± 0.009^c^	4.55 ± 0.79^c^	27.81 ± 2.79^bc^
0.7%BOEO	0.070 ± 0.004^a^	2.93 ± 0.75^c^	32.15 ± 2.34^b^
1.4% BOEO	0.064 ± 0.008^b^	2.35 ± 0.05^c^	25.00 ± 1. 31^bc^
2.1% BOEO	0.060 ± 0.007^c^	2.16 ± 0.13^c^	14.01 ± 2.23^c^

*Note:* The results were expressed as mean value ± SD (*n* = 3). Different letters within the same figure mean statistical difference (*p* < 0.05).

### 3.2 Water Vapor Permeability, Contact Angle, Solubility, and Water Absorption of Bilayer PLA‐Chitosan/Starch Films Containing BOEO

As shown in Table 4, the addition of chitosan increased the contact angle values, solubility, and water absorption of the films, which may be attributed to interactions between these polymers, such as electrostatic forces and hydrogen bonding. The water vapor permeability values decreased, which could be due to the chitosan film having a higher water‐repellent property (lower water affinity) compared to starch, ultimately resulting in reduced water vapor permeability. Furthermore, with the addition of BOEO to the bilayer film matrix, the water vapor permeability, solubility, and water absorption values decreased. This reduction may be due to increased water vapor passage, which can be attributed to the arrangement and compounds present in bitter orange causing changes in the surface molecular structure of the polymer. As a result, the surface structure density decreases, accelerating the passage of water vapor from within the films. Additionally, the contact angle values increased, which is due to the water‐repellent nature of the added bitter orange essential oil in the films, preventing water from entering the film structure (Zhang et al. 2021).

**TABLE 4:** The results of contact angle at initial time, water vapor permeability, solubility, and water absorption of bilayer PLA‐chitosan/starch films containing BOEO.

Treatments	Contact angle at initial time (°)	Water vapor permeability (g mm kPa h m^2^)	Solubility (%)	Water absorption (%)
100PLA/0CHST	75.36 ± 2.64^ab^	0.30 ± 0.08^d^	6.17 ± 2.70^g^	1.44 ± 0.22^f^
75PLA/25CHST	68.67 ± 1.52^b^	0.19 ± 0.05^e^	88.17 ± 3.16^a^	58.08 ± 4.01^d^
50PLA/50CHST	82.72 ± 2.83^a^	0.55 ± 0.03^cd^	74.95 ± 4.10^b^	92.1 ± 4.88^c^
25PLA/75CHST	23.4 ± 1.67^c^	0.79 ± 0.01^c^	53.05 ± 2.19^d^	135.63 ± 3.4^b^
0PLA/100CHST	61.53 ± 2.33^b^	0.33 ± 0.00^d^	20.96 ± 1.84^f^	181.2 ± 5.18^a^
0.7% BOEO	22.33 ± 1.29^c^	0.16 ± 0.01^e^	64.29 ± 3.36^c^	50.99 ± 3.33^d^
1.4% BOEO	35.45 ± 2.94^c^	0.135 ± 0.00^b^	50.25 ± 2.46^d^	40.31 ± 3.20^e^
2.1% BOEO	70.1 ± 2.81^b^	0.184 ± 0.06^a^	39.87 ± 2.60^e^	39.88 ± 3.96^e^

*Note:* The results were expressed as mean value ± SD (*n* = 3). Different letters within the same figure mean statistical difference (*p* < 0.05).

### 3.3 Colorimetric Results of Polylactic Acid/Chitosan: Starch Bilayer Films Containing Different Concentrations of BOEO

The results of colorimetric properties show that the formation of bilayer films has caused a significant change in the b* index (blue‐yellow) compared to single‐layer films (Table 5). Regarding the L* index (brightness), it can be said that the formation of bilayer films had a significant impact on film transparency, in which starch films and composite films (25% and 50% starch) appeared yellower compared to chitosan films. This can be attributed to the natural color and nature of starch, which absorb light in the lower wavelengths. With an increase in the amount of BOEO in the polylactic acid/chitosan: starch system, a significant difference in the L* and a* factors was observed at the 1/2% level in that the highest b* value (yellow) was observed in the polylactic acid/chitosan:starch film containing 1/2% BOEO. Color variations and color indices decreased significantly with an increase in oil content. However, as the amount of bitter orange oil increased up to the 1/2% level, the whiteness index significantly improved. The cause of this phenomenon can be attributed to the components of BOEO, as phenolic compounds present in this oil are capable of absorbing light in the lower wavelengths.

**TABLE 5:** The results of color parameters of bilayer PLA‐chitosan/starch films containing BOEO.

Treatments	Color parameters				
	L*	α*	b*	ΔE	WT
100PLA/0CHST	39.01 ± 0.93^c^	3.5 ± 2.25^b^	1.75 ± 1.59^a^	55.85 ± 0.69^a^	38.84 ± 0.73^b^
75PLA/25CHST	37.00 ± 1.49^c^	6.95 ± 0.62^a^	−1.39 ± 0.35^c^	58.20 ± 1.49^a^	36.59 ± 1.49^b^
50PLA/50CHST	41.84 ± 1.79^c^	6.62 ± 0.32^a^	−0.70 ± 0.37^c^	53.34 ± 1.81^a^	41.45 ± 1.81^b^
25PLA/75CHST	39.47 ± 2.16^c^	7.03 ± 0.89^a^	−0.95 ± 1.03^c^	55.75 ± 2.10^a^	39.04 ± 2.11^b^
0PLA/100CHST	46.61 ± 1.35^b^	6.13 ± 0.42^a^	0.58 ± 0.40^b^	48.53 ± 1.40^a^	46.25 ± 1.39^b^
0.7%BOEO	82.04 ± 0.10^a^	0.25 ± 0.09^c^	1.77 ± 0.15^a^	12.68 ± 0.09^b^	81.95 ± 0.08^a^
1.4% BOEO	82.11 ± 0.15^a^	0.18 ± 0.01^c^	1.82 ± 0.05^a^	12.61 ± 0.14^b^	82.01 ± 0.14^a^
2.1% BOEO	82.38 ± 0.07^a^	0.07 ± 0.05^c^	1.96 ± 0.02^a^	12.35 ± 0.07^b^	82.27 ± 0.07^a^

*Note:* The results were expressed as mean value ± SD (*n* = 3). Different letters (a, b, c) within the different treatments mean statistical difference * (*p* < 0.05).

### 3.4 Scanning Electron Microscopy (SEM)

The results of Scanning Electron Microscopy are shown in Figure 1a. The SEM images of the ST:100CH0 and ST:0CH100 films show a relatively smooth surface devoid of pores and cracks. As the starch content increased from 25% to 75%, a different surface arrangement was observed in the bilayer films, resembling a rough and flaky structure. This phenomenon may result from the orientation of polar functional groups towards the upper surface of the composite films. The cross‐sectional images of chitosan films, starch films, and chitosan/starch films exhibited continuous homogeneous morphology with a dense structure and no irregularities such as bubbles or pores, phase separation, which might be due to intermolecular bonding between polymers through hydrogen bonding and good compatibility between chitosan and starch polymers, leading to improved phase stability. SEM observations indicated that the 25PLA/75CHST film was nearly smooth, uniform, and without any bubbles, pores, or cracks (Figure 1b). The surface view and cross‐sectional images of films containing BOEO indicated some changes in the polymer structure, such as the formation of pores, discontinuities, and rounding of polymer particles. Additionally, the uniform distribution of oil droplets within the polymer matrix is visible. The observations suggested that the size of oil droplets increased with an increase in BOEO concentration. At high concentrations of the antimicrobial substance (1/2%), the increased volume of oil droplets in the film solution leads to changes in the polymer chains, resulting in particle rounding and the opening of spaces within the matrix, ultimately leading to increased roughness, decreased tensile strength, and reduced water vapor permeability of the produced films. A similar investigation was published by Javidi et al. (2016), in which they produced a poly lactic acid film with essential oil.

### 3.5 Atomic Force Microscope (AFM)

The results obtained from atomic force microscopy (AFM) show that with an increase in the chitosan content, the surface roughness declined (Figure 2). The reduction in roughness of the films may be due to the interaction between the two polymers, chitosan and starch, which is related to the arrangement and type of molecular chains and the amount of empty space between them in the polymer matrix (Chen et al. 2024). Additionally, electrostatic forces between the molecules of the two polymers play a role in creating these structural differences. However, the results of adding bitter orange oil showed that with an increase in oil concentration, the surface roughness of the films increased. Films containing bitter orange essential oil exhibited more non‐uniformity compared to the control film. This phenomenon might be due to the accumulation of oil droplets after drying on the film surface, creating surface matrix irregularities (Javidi et al. 2016). Javidi et al. (2016) similarly reported that adding *Origanum vulgare* L. essential oil in PLA‐based film led to an increase in their roughness. On the other hand, using ginger essential oil declined the surface roughness of chitosan/polyvinyl film‐based film (Chen et al. 2024).

### 3.6 Thermal Behavior

Differential scanning calorimeter (DSC) of the prepared films is shown in Figure 3. Two endothermic peaks were observed for all films; the first endothermic peak in the temperature range from 43.2°C to 71.9°C is attributed to phenomena such as the fixing of absorbed water, residual acetic acid, polymer degradation, and the melting temperature of the polymer. Meanwhile, the second peak in the temperature range from 87.4°C to 163.4°C indicated thermal decomposition due to the pyrolytic decomposition of the chitosan structure. With an increase in the amount of chitosan in the bilayer films, the melting point (Tm) and thermal stability of the films decreased, which is in line with the increase in free volume of polymers and their mobility. Moreover, by adding BOEO to the PAC‐based film matrix, the melting temperature (Tm) and glass transition temperature (Tg) of the samples slightly increased. These observations indicate that the addition of bitter orange oil enhanced the thermal stability of the prepared films, which might be due to the interaction of orange oil and chitosan and leading to a higher crystalline structure (Pankaj et al. 2014). This result is aligned with the investigation of Liu et al. (2024a) when they reported that the presence of essential oils improved the thermal properties of chitosan‐based composite film. Additionally, it is proven that adding 10% citronella and cedarwood essential oils can increase the thermal stability of chitosan film (Shen and Kamdem 2015).

### 3.7 Fourier Transfer Infrared (FTIR)

**FIGURE 4:**
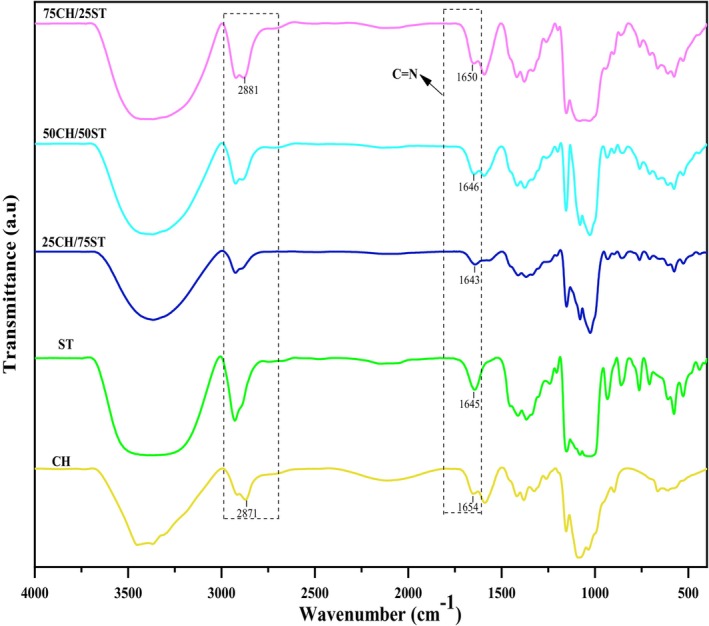
FTIR spectrum of the chitosan:Starch composite films and polylactic acid/chitosan:Starch bilayer films, and polylactic acid/chitosan:Starch films containing concentrations of 0.7%, 1.4%, and 2.1% BOEO.

The chemical bonds of chitosan‐based films are illustrated in Figure 4. In the spectrum of chitosan film, a strong peak in the range of 3351 cm^−1^ is attributed to the stretching vibrations of O‐H groups, which overlap with the stretching vibrations of N‐H groups in the same range (Chen et al. 2024). Additionally, the observed peak at 1578 cm^−1^ may be associated with the N‐H (amide II) bending. Two small peaks at 1655 cm^−1^ and 1741 cm^−1^ were assigned to the stretching vibrations of C=O (amide I) and the presence of carbonyl groups, respectively (Ji et al., 2017). In the spectrum of the chitosan film, a strong peak in the range of 3351 cm^−1^ is attributed to the stretching vibrations of O‐H groups, which overlap with the stretching vibrations of N‐H groups in the same range (Chen et al. 2024). After incorporating bitter orange oil, some of these peaks in the spectrum of the PC‐based film containing 1.2% BOEO shifted to higher or lower wavelengths. In the PC‐based film containing 1.2% of BOEO, an increase in alkyl groups (CH, CH2, and CH3) in the range of 2860 cm^−1^ to 2990 cm^−1^ indicated the hydrophobicity rising. Additionally, new peaks appeared in the wavelength range between 1400 cm^−1^ and 1600 cm^−1^, which could be related to the presence of several saturation of aromatic hydrocarbons such as carvacrol, thymol, p‐cymene, γ‐terpinene, and phenolic compounds in BOEO. Therefore, the addition of BOEO altered the molecular composition and intermolecular interactions in the film matrix, resulting from the intermolecular interaction between chitosan and BOEO. The result was aligned with the investigations of Mehdizadeh et al. (2020) and Chen et al. (2024).

### 3.8 Antioxidant Analysis

**FIGURE 5:**
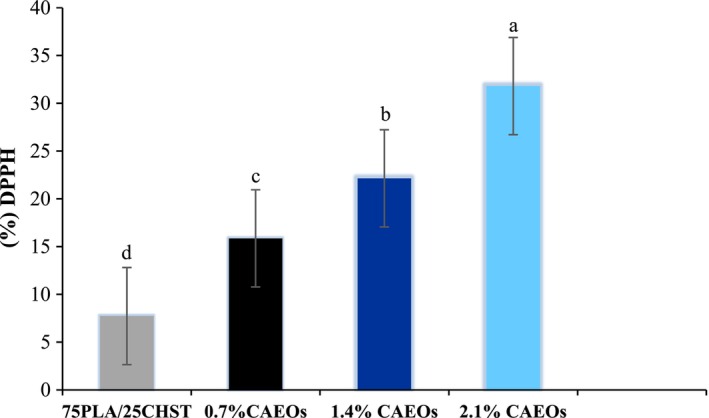
DPPH radical scavenging activity of polylactic acid/chitosan:Starch films containing concentrations of 0.7%, 1.4%, and 2.1% BOEO.

DPPH radical scavenging activity of PC‐based films is illustrated in Figure 5. As can be seen, adding BOEO resulted in a significant increase in the antioxidant capacity of PC‐based films. The highest antioxidant ability was observed at 2.2% BOEO, while the lowest was recorded in the 75PLA/25CHST formulation. The inferior antioxidant activity of the bilayer film can be attributed to the reaction between free radicals and the remaining free amine groups from the ammonium group, while the enhanced antioxidant activity of BOEO‐containing films might be due to the presence of eugenol and the synergistic effects of the available polymers, like eugenol and BOEO. Similarly, Hafsa et al. (2016) proved that using EG essential oil can incredibly increase the antioxidant properties of chitosan‐based film. Moreover, Kamkar et al. (2021) reported that adding nano‐liposomal essential oil improved the antioxidant activity of chitosan‐based film.

### 3.9 Antimicrobial Analysis

Antimicrobial analysis of the produced films against different Gram‐positive and Gram‐negative bacteria is reported in Table 6. The 75PLA/25CHS formulation showed no bacterial growth, which could be attributed to the intrinsic antimicrobial properties of chitosan. The composite film containing 0.7% BOEO was able to completely inhibit *L. monocytogenes* , *S. enteritidis* , *E. coli* , and to some extent, *S. aureus* . However, the bilayer film containing 1.4% and 2.1% BOEO showed a good but lower inhibitory effect against bacteria: *L. monocytogenes* , *S. enteritidis* , *E. coli* , and *S. aureus* . The antimicrobial inhibitory effect of BOEO‐rich films against bacteria can be due to the presence of active components in essential oils which can be volatile into the atmosphere. The lower inhibitory effect of 1.4% and 2.1% BOEO‐contained films can be justified by the fact that chitosan declines the release speed of essential oils through the interaction between its polymers and essential oils, resulting in a slower but long‐lasting inhibitory effect (Li et al., 2019). Another factor could be the presence of lipopolysaccharides in Gram‐negative bacteria, which may obstruct the entry of active compounds into the cytoplasmic membrane. Sánchez‐González et al. (2010) reported that chitosan‐bergamot essential oil films indicated superior inhabitation ability against the growth of *Penicillium italicum*. In another study, Liu et al. (2024b) proved that adding turmeric essential oil in a chitosan film enhanced the inhibitory effect of the film against *Aspergillus flavus*. Research using *Mentha longifolia* essential oil in electrospun food packaging films reported by Shahbazi et al. (2021) shows that natural essential oils can significantly extend the shelf life of seafood. This confirms earlier findings with bitter orange essential oil (BOEO) in similar biodegradable films. These films, created using electrospinning, not only improve the packaging's strength and barrier properties but also allow for the controlled release of the antimicrobial essential oil, thereby inhibiting bacterial growth and improving food safety. Our work with BOEO‐enhanced films for rainbow trout further supports the effectiveness and sustainability of this approach to seafood preservation.

**TABLE 6:** Antimicrobial properties of double‐layered films, double‐layered films of polylactic acid/chitosan: Starch containing different concentrations of BOEO against some positive and negative gram bacteria.

The type of film	*L. monocytogenes*	*S. enteritidis*	*E. coli*	*S. aureus*
75PLA/25CHST	0	0	0	0
0.7%CAEOs	0	0	0	11.21 ± 0.11^c^
1.4% CAEOs	0.36^b^ ± 21.5	46.9 ± 0.41^a^	19.8 ± 0.19^b^	97.28 ± 0.34^b^
2.1% CAEOs	48.9 ± 0.51^a^	117.6 ± 0.32^b^	43.4 ± 0.21^a^	124.5 ± 0.53^a^

*Note:* The results were expressed as mean value ± SD (*n* = 3). Different letters within the same figure mean statistical difference (*p* < 0.05).

### 3.10 The Results of the Changes of Some Microbial Indicators of Fillet Covered With Prepared Film During Storage

#### 3.10.1 Counting the Total Bacterial Load (TVC)

The content of TVC during storage (16 days) is represented in Table 7; the initial total bacterial load of the fish used was found to be 2/85 log_10_ CFU/g, indicating good quality of the prepared fish. Over time, the level of TVC increased in samples, with the highest TVC observed in the final days of the storage period and the lowest amount seen on day zero. The sample covered with PC‐based film containing 1.2% BOEO exhibited slower decay compared to other treatments, which was indicated by the lowest TVC amount during storage, attributed to the desirable inhibitory properties of the bitter orange essential oil. The highest ability of this film for spoilage delaying is attributed to its phenolic compounds, including carvacrol and thymol, which can protect bioactive compounds against degradation (Pabast et al., 2018). This result was in harmony with the study of Lv et al. (2018) in which they showed that the utilization of polyethyleneimine (PEI) and thyme essential oil (T) in chitosan film delayed the microbial spoilage of sliced fresh *Channa argus* during storage with a lower amount of TVC. Additionally, Valipour Kootenaie et al. (2017) reported that using eucalyptus essential oil in chitosan‐based film delayed the rate of TVC growth on silver carp during 16 days of storage. The study conducted by Rezaei and Shahbazi (2018) provides valuable insights into the effectiveness of different preservation methods in controlling bacterial growth and extending the shelf life of fish products. Similar to our findings on the use of bitter orange essential oil (BOEO) in PLA/chitosan films, this study demonstrated that biodegradable films were more effective than direct addition or edible coatings in reducing spoilage bacteria such as psychrotrophic and lactic acid bacteria. This highlights the superior barrier properties and controlled release of antimicrobial agents achieved through film‐based packaging. The results align with our observations that BOEO‐incorporated films significantly reduced bacterial counts in rainbow trout fillets during refrigerated storage, emphasizing the role of innovative packaging in maintaining seafood quality and safety.

**TABLE 7:** The results of bacterial and pH change of coated fish fillets by PLA‐chitosan/starch films containing BOEO.

	Treatments	Storage days				
		0	4	8	12	16
TVC	Control	2.85 ± 0.46^a^	4.12 ± 0.19^a^	7.17 ± 0.41^a^	8.99 ± 0.28^a^	10.82 ± 0.24^a^
	75PLA.25CHST	2.85 ± 0.46^a^	3.84 ± 0.13^b^	6.40 ± 0.26^ab^	8.62 ± 0.19^ab^	10.39 ± 0.35^b^
	2.1% BOEO	2.85 ± 0.46^a^	3.17 ± 0.19^c^	4.70 ± 0.32^b^	5.79 ± 0.22^b^	9.15 ± 0.29^b^
PTC	Control	3.07 ± 0.03^a^	4.73 ± 0.13^a^	7.62 ± 0.11^a^	8.75 ± 0.21^a^	10.12 ± 0.16^a^
	75PLA.25CHST	3.07 ± 0.04^a^	4.51 ± 0.14^ab^	7.63 ± 0.23^a^	8.12 ± 0.13^ab^	10.02 ± 0.27^a^
	2.1% BOEO	3.07 ± 0.05^a^	3.75 ± 0.19^b^	7.05 ± 0.13^b^	8.79 ± 0.22^c^	9.01 ± 0.13^b^
Lactic acid bacteria	Control	1.98 ± 0.18^a^	3.31 ± 0.14^a^	5.15 ± 0.17^a^	6.18 ± 0.09^a^	6.48 ± 0.24^a^
	75PLA.25CHST	1.98 ± 0.18^a^	3.22 ± 0.32^b^	5.26 ± 0.19^a^	6.03 ± 0.08^a^	6.32 ± 0.27^a^
	2.1% BOEO	1.98 ± 0.18^a^	2.28 ± 0.13^a^	3.12 ± 0.16^b^	4.18 ± 0.13^b^	4.51 ± 0.41^b^
Enterobacteriaceae bacteria	Control	2.11 ± 0.05^a^	4.06 ± 0.11^a^	5.81 ± 0.17^a^	7.96 ± 0.14^a^	8.17 ± 0.20^a^
	75PLA.25CHST	2.11 ± 0.05^a^	3.75 ± 0.08^b^	5.77 ± 0.12^a^	7.35 ± 0.13^b^	8.06 ± 0.15^a^
	2.1% BOEO	2.11 ± 0.05^a^	2.92 ± 0.07^c^	3.99 ± 0.16^b^	4.92 ± 0.41^c^	6.99 ± 0.21^b^
pH values	Control	6.63 ± 0.00^a^	6.36 ± 0.03^a^	6.88 ± 0.04^a^	7.00 ± 0.24^a^	7.41 ± 0.13^a^
	75PLA.25CHST	2.11 ± 0.00^b^	6.22 ± 0.11^a^	6.44 ± 0.11^b^	6.62 ± 0.19^b^	7.29 ± 0.35^ab^
	2.1% BOEO	2.11 ± 0.00^b^	2.27 ± 0.23^b^	6.21 ± 0.11^c^	6.38 ± 0.22^c^	6.94 ± 0.29^b^

*Note:* The results were expressed as mean value ± SD (*n* = 3). Different letters within the same figure mean statistical difference (*p* < 0.05).

#### 3.10.2 Counting the Psychrotrophic Bacteria (PTC)

The level of Gram‐negative psychrotrophic bacteria (PTC) in fish during storage is reported in Table 7. The initial psychrotrophic bacteria count for all treatments on day zero was 3.07 log_10_ CFU/g, which increased during storage for all samples. Even though all coated samples indicated acceptable levels for consumption on day 8, the sample coated with PC‐based film containing 2.1% BOEO had the lowest amount of PTC on the mentioned day of storage compared to other samples, resulting from the inhibitory effect of phenolic compounds, which have a great impact on the function of bacterial enzymes, resulting in the leakage of intracellular constituents (Ouattara et al. 2000). Ojagh et al. (2010) indicated that adding cinnamon oil to chitosan film delayed the growth of Gram‐negative psychrotrophic bacteria in rainbow trout.

#### 3.10.3 Counting the Lactic Acid Bacteria

The results showed an increasing trend in the lactic acid bacteria (LAB) count in all treatments over the storage period (Table 7). The growth rate of LAB was found to be similar in both the control and the sample covered with PC‐based film, while the fillets coated with PC‐based film containing h 1.2% BOEO showed a lower level of LAB during the storage, revealing the positive synergistic effect of chitosan and BOEO for maintaining the quality of fish. Cai et al. (2019) revealed that the utilization of lemon and thyme essential oils in chitosan film reduced the growth rate of LAB on grass carp fillets. Using *Z. multiflora* essential oils along with chitosan was also found to be effective for slowing the growth of LAB in rainbow trout fillets (Raeisi et al. 2020).

#### 3.10.4 Counting the Enterobacteriaceae Bacteria

The initial levels of Enterobacteriaceae bacteria on the first day of storage for all treatments were 2.11 log_10_ CFU/g, which increased over time, with a more intense increase observed in the uncovered treatment and PC‐based film compared to the treatments containing BOEO (Table 7). By day 16, the levels of Enterobacteriaceae bacteria in these treatments were 8.17 log_10_ CFU/g in control, 06.8 log_10_ CFU/g in 75PLA.25CHST, and 99.6 log_10_ CFU/g in CAEos 2.1%. This phenomenon can be attributed to the protective effect of the BOEO against these bacteria, as it contains numerous phenolic compounds, which inhibit the growth of bacteria. Moosavi‐Nasab et al. (2016) suggested that using chitosan coating enriched with black pepper essential oils can reduce the growth rate of Enterobacteriaceae bacteria in stored common carp. Moreover, Cai et al. (2019) showed that using lemon and thyme essential oils in chitosan coating can reduce the level of Enterobacteriaceae bacteria in grass carp fillets during storage.

### 3.11 pH Level

Changes in pH level are represented in Table 7. The results indicated pH changes over time in all treatments, with the pH level decreasing from 6.36 on day zero to 4.17 on day 16 in the control sample. The pH changes in the control sample were more noticeable than those in other treatments, ending with higher pH levels at the end of the storage. Meanwhile, the level of pH in the sample covered with 2.1% CAEOs reached 9.46 at the end of the period. A decrease in pH was observed at the beginning of the storage, which can be considered the result of CO_2_ dissolution from glycogen breakdown in fish tissues and the formation of carbonic acid. The increase in pH in the control and covered treatments can be attributed to the increased production of volatile compounds such as ammonia and trimethylamine due to bacterial enzymatic activities. The lower pH in fillets covered with films BOEO throughout the storage can be due to the potential inhibitory effect of phenolic compounds that are available in BOEO on bacterial activities and enzymatic proteases. Kamkar et al. (2021) announced that using nano‐liposomal essential oil along with 1% chitosan can decline the level of pH in chicken breast fillet. Zomorodian et al. (2023) recommended that using chitosan film containing ZM‐PE reduced the pH level in salmon. Majidiyan et al. (2022) reported that incorporating industrial hemp essential oil effectively slowed down the increase in pH, maintaining the freshness of the fish fillets over time. Similarly, in our study, using PLA/chitosan films containing bitter orange essential oil (BOEO) significantly inhibited the rise in pH levels during refrigerated storage. Both studies demonstrate that natural essential oils can suppress microbial activity and reduce the production of basic nitrogenous compounds, which are responsible for pH elevation in fish products.

### 3.12 TBARS Level

**FIGURE 6:**
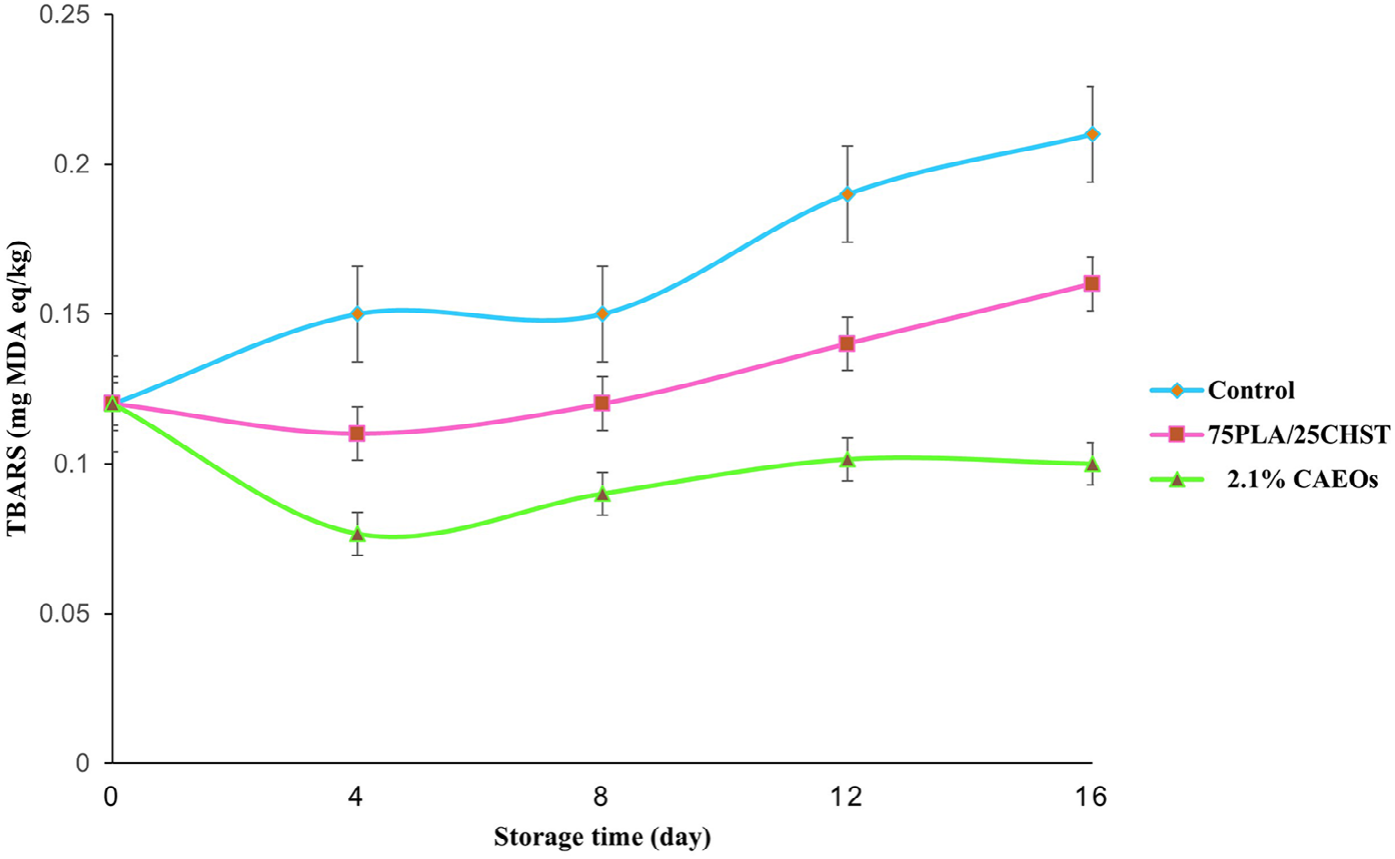
TBA changing of the fish fillet during storage in the refrigerator.

The level of TBARS during 16 days is illustrated in Figure 6. The results indicated that the level of TBA was low in all samples on the first day of storage, which increased slowly until the last day. This increasing trend in the level of TBA can be related to the secondary oxidation spoilage of fats. The highest level of TBA was observed in the control sample, while the lowest level was related to the sample covered with 2.1% CAEos during the whole duration of storage. The lowest TBA level in this sample might be due to the oxidative properties of BOEO. Kamkar et al. (2021) proved that nano‐liposomal essential oil in chitosan film indicated an excellent inhibitory effect against TBARS in chicken breast fillets. It was also shown by Zomorodian et al. (2011) that the chitosan film enriched with *Zataria multiflora* essential oils was able to reduce the level of TBARS in salmon. Shahbazi et al. (2021) reported a significant reduction in prawn samples packaged with *Mentha longifolia* essential oil films throughout the storage period. This confirms the antioxidant properties of the essential oil and its effectiveness in inhibiting lipid peroxidation. This finding aligns with our research on bitter orange essential oil (BOEO) in PLA/chitosan films, where we also observed a significant reduction in TBARS values in rainbow trout fillets. Both studies demonstrate the potential of incorporating natural essential oils into packaging materials to enhance oxidative stability and extend the shelf life of fish products, maintaining sensory quality and reducing food waste.

## 4 Conclusion

In this study, we investigated the development and application of biodegradable films based on polylactic acid (PLA) and chitosan:starch, enhanced with bitter orange essential oil (BOEO), for the preservation of rainbow trout fillets. Our findings contribute significant advancements to the field of food packaging and preservation, highlighting several novel aspects of our approach. Firstly, the incorporation of BOEO into the PLA/chitosan:starch films demonstrated a marked improvement in antimicrobial activity. The optimal concentration of 1.2% BOEO effectively inhibited both Gram‐positive and Gram‐negative bacteria, which are known to contribute to spoilage in seafood products. This novel application of natural essential oils as an antimicrobial agent not only enhances food safety but also aligns with consumer preferences for natural and non‐toxic preservatives. Secondly, our study revealed that these films significantly reduced spoilage indicators in rainbow trout fillets during refrigerated storage. By slowing the increase in pH and minimizing TBA values, we provided evidence that BOEO‐infused films can maintain the quality of fish products over an extended period. This finding is particularly relevant in addressing the challenge of maintaining freshness in perishable goods, thus offering a practical solution for the seafood industry. Moreover, we explored the mechanical properties and structural characteristics of the films using advanced techniques such as atomic force microscopy (AFM) and scanning electron microscopy (SEM). The results indicated that the addition of BOEO not only altered the surface morphology but also enhanced film hydrophobicity and thermal resistance, providing insights into how these properties can be optimized for better performance in food packaging applications.

## Author Contributions

H.N.: Investigation, methodology, formal analysis, writing – original. S.M.O.: Supervision, project administration, conceptualization, resources, writing – review and editing. M.R.: Supervision, project administration, investigation, writing – review and editing. A.A.: Conceptualization. S.N.: Conceptualization, writing – review and editing.

## Conflicts of Interest

The authors declare no conflicts of interest.

## Data Availability

The data that support the findings of this study are available on request from the corresponding author. The data are not publicly available due to privacy or ethical restrictions.
